# What We Have Learned and What We Have Missed in Tuberculosis Pathophysiology for a New Vaccine Design: Searching for the “Pink Swan”

**DOI:** 10.3389/fimmu.2017.00556

**Published:** 2017-05-15

**Authors:** Pere-Joan Cardona

**Affiliations:** ^1^Unitat de Tuberculosi Experimental, Institut Germans Trias i Pujol, Crta de Can Ruti s/n, Badalona, Catalonia, Spain

**Keywords:** *Mycobacterium tuberculosis*, bacillus Calmette–Guerin vaccine, pink swan, dynamic hypothesis, *Mycobacterium tuberculosis* tolerance, damage theory

## Abstract

This is a call to encourage the search for a new vaccine to stop the progression of *Mycobacterium tuberculosis* infection to tuberculosis (TB) disease. TB is a highly discreet and stigmatized disease, with a massive impact on human health. It has killed 1.2 billion people in the last 200 years and still kills 1.5 million people per year. Over the last 20 years, the TB vaccine field has experienced spectacular developments, and we have learned about (1) the importance of the Th1 response in controlling infection, mainly against RD1 and Ag85 antigens; (2) the stability of the antigenic repertoire; (3) the dynamics of *M. tuberculosis* granulomas; or (4) the link between typical and atypical pulmonary TB and the immune status of the host. However, we still do not (1) know how to avoid *M. tuberculosis* infection and reinfection; (2) understand the major role of the increase in lesion size in progression from infection to disease; (3) the role of interlobular septa in encapsulating pulmonary lesions; or (4) the role of neutrophilic infiltration and an exaggerated inflammatory response in the development of TB disease. These are strong reasons to pursue new, imaginative proposals involving both the antibody response and a balanced, tolerant immune response that averts progression toward TB. So far, the scientific mindset has been quite monolithic and has mainly focused on the stimulation of conventional T cells. But this approach has failed. For that reason, we are seeking unconventional perspectives to find a “pink swan,” a more efficacious and safer vaccine candidate.

## Tuberculosis Control: A Failed Prophecy

Discovery consists of seeing what everybody has seen, and thinking what nobody has thought—Albert Szent-György.

In 1993, the WHO declared tuberculosis (TB) a global emergency. The old, optimistic prophecy that had predicted its eradication by the year 2000 did not materialize. This enthusiasm was mainly due to the short-term chemotherapy developed in the 1980s. Alongside relatively inefficient case detection, the problem was the relative nature of the “short-term” concept, meaning 6 months and the fact that treatment required the administration of at least three different chemotherapeutic drugs. This approach needed a certain level of organizational support, such as the Directly Observed Therapy (DOTS) strategy that was not always achieved, for various reasons, and which, together with a lack of patient compliance due to the lengthy, cumbersome treatments, led to the generation of multidrug resistant strains (1), with some *Mycobacterium tuberculosis* sublineages being more prone to developing resistance than others (2). The irruption of the HIV epidemic also had a substantial impact on the induction of new TB cases. Finally, the increase in human mobility around the planet has linked high-incidence areas with low-incidence ones, while growing urbanization, with the inherent rise in crowding, has done the rest (3).

One aggravating problem is TB’s insidious nature, not only due to the clinical course of the disease but also because of the stigma linked to TB that leads patients and their families to hiding it (4). In consequence, social awareness of the magnitude of the problem of TB diminishes, leading to a reduction in the investments needed to defeat it.

Another reason for TB’s elusiveness is that infection is totally asymptomatic and harmless and does usually not progress toward disease. Hence, in TB, it is very important to distinguish between infection and disease. Infection is caused by the inhalation of aerosols with *M. tuberculosis*, which reach the alveolar space and infect alveolar macrophages. Nevertheless, this infection only causes disease in a minority of cases, and the risk decreases exponentially over time, being the highest (80%) during the first 2 years after challenge (5). This is why there are no “explosive” TB epidemics over short-time periods, decreasing its visibility and reducing stakeholders’ and politicians’ interest in it. To tackle TB, short-term interventions and short-term solutions are far from being the answer.

Tuberculosis is the most underestimated major killer of humankind. TB has caused 1.2 billion deaths over the last 200 years. Even nowadays, in the twenty-first century, TB caused 1.5 million deaths and 9 million new cases, 480,000 of which were MDR-TB, in 2016 (6). Most importantly, a third of these are not even detected, a proportion that reaches 50% in some areas, especially in Asia and Africa (6).

## The Quest for a New Vaccine Against TB

Immediately after the declaration of the global emergency, the idea of having a more efficacious TB vaccine became a major goal. The Bacillus Calmette–Guerin (BCG) vaccine has been available since 1927. It is the world’s most widely used vaccine and has been administered massively to neonates, with over 3 billion vaccinations performed so far (7). BCG can avoid the development of quickly progressing, deadly forms of the disease such as meningitis and disseminated (miliary) TB, but it neither avoids infection nor lung disease. In addition, BCG revaccination of teenagers, at a time when the effect of the first administration has dissipated, does not offer any additional protection to the population (8).

The enormous effort made over the last 20 years, seeking for a new vaccine based on a conventional T cell response, appears to have been of limited value. For that reason, the idea of searching for a “pink swan” is spreading in the TB field. It is inspired by the concept of “black swan,” used in the field of economics by N.N. Taleb referring to an unpredictable event with massive, ground-breaking consequences. The “pink swan” illustrates a fresh, unconventional approach, driven by a currently unknown mechanism that will bring us a new, more efficacious and safer vaccine (9).

### What We Have Learned So Far

#### The Th1 Cellular Immune Response: The Mouse Model

The reappraisal of TB vaccine development in the 1990s coincided with the availability of new, powerful tools for cellular immune analysis, like the flow cytometer, and the technology needed to generate knockout (KO) mice. Thanks to this, the paramount role of IFN-γ in the control of *M. tuberculosis* infection was soon demonstrated. IFN-γ-KO mice were the ones with the lowest survival ratio (10).

The experimental mouse model gained a lot of relevance and became the first gateway for every new candidate for vaccine development, while BCG became the positive gold standard. Mice vaccinated with BCG showed a reduction of around 0.7 logs in colony-forming units in the lung 3 weeks after challenge, when the highest bacillary load was reached. This was determined in inbred, immunocompetent mice, usually C57BL/6 (11).

It led to a search for vaccines able to induce a Th1 response against *M. tuberculosis* antigens. In fact, all vaccines currently in clinical trials were selected on the basis of their capacity to induce Th1 biased CD4 T cells and, to a lesser extent, on CD8 T cell responses (12). Nevertheless, it soon became evident that *M. tuberculosis* infection could, in itself, induce a protective Th1 immune response, at least as effective as the one induced by BCG vaccination. This prompted the need to look for candidates able to induce “something else” to ensure protection against *M. tuberculosis* infection (13).

This “something else” could be multifunctional T cells. These cells have a prolonged memory and effector potential and can produce not only IFN-γ but also IL-2 and TNF-α, reinforcing the surveillance against *M. tuberculosis*. The protective effect of these cells was described in the *Leishmania* model, where their presence was linked to a significant reduction in the number of lesions and amastigotes (14).

Unfortunately, in the majority of cases, the presence of these cells did not appear to make a difference in TB disease development between protected and non-protected subjects (15). They can be considered a marker determining the presence of long-lasting memory cells, but this does not mean that they have to be protective. There are still several authors delving into this issue. Recently, it has been found that multifunctional cells specific for DosR and Rfp antigens can be linked to protection after using a long-term culture assay (16).

#### Antigenic Discovery: The RD1-Encoded Antigen and the Ag85 Complex Proteins

At the beginning of the 1990s, two groups of *M. tuberculosis* antigens, the ones triggering the greatest Th1 response, gained great relevance. As dead bacilli were not able to induce a protective response in the mouse model, attention was turned to the proteins secreted by active growing bacilli (17). It was soon determined that there were two groups of proteins capable of triggering the greatest Th1 response. These were, on the one hand, the RD1-encoded antigens, mostly linked to the production of ESAT-6 and its secretion *via* its carrier: CFP-10. ESAT-6 is responsible for inducing the lysis of host cell phagosomes, thus being a paramount virulence factor (18). On the other hand, the Ag85 complex, which has a major role in the construction of the cell wall *via* its mycolyltransferase activity (19).

#### The Genomic Sequence

*Mycobacterium tuberculosis* sequencing is one of the milestones in the field of TB research. It has allowed a better comprehension of the physiology of *M. tuberculosis* and its regulatory mechanisms against stress (20). It has also been a key to a better understanding of the origin and evolution of the species as well as the characterization of the different geographical families, linked in turn to the evolution of humankind (21). Interestingly, sequencing has also shown the extraordinary stability of the antigenic repertoire and the immune response against this repertoire, which does not vary according to the different families defined (22).

#### Granuloma Dynamics

**Figure 1:**
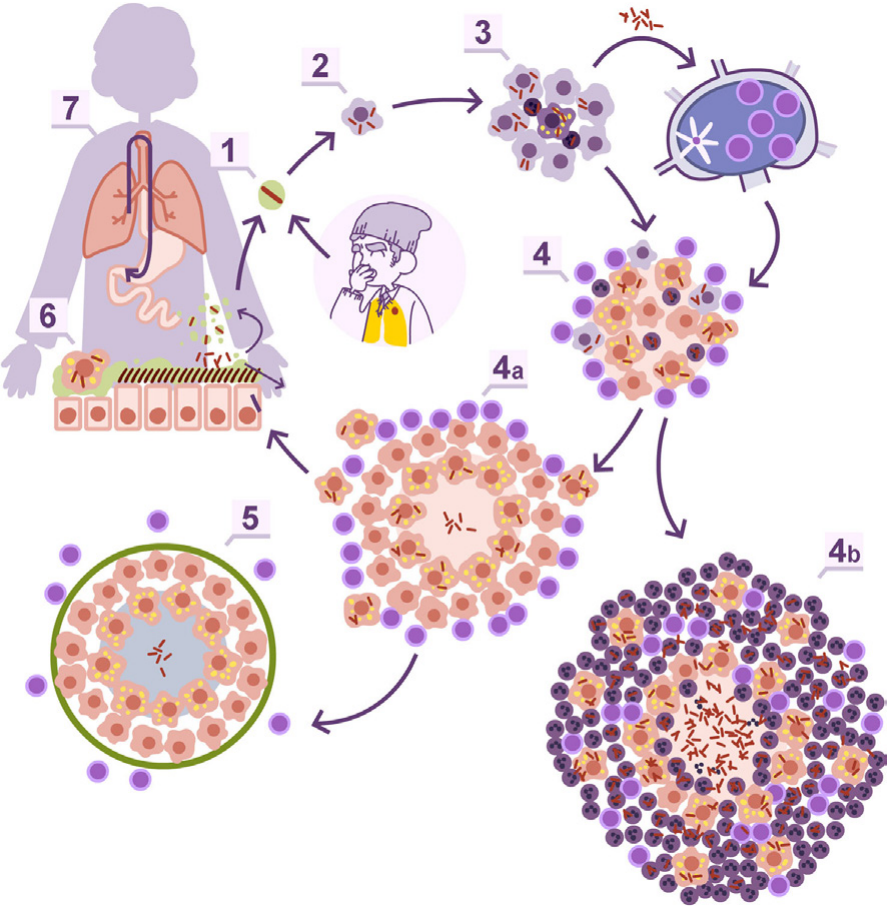
**Dynamic hypothesis of *Mycobacterium tuberculosis* infection**. Bacilli contained in an aerosol droplet of endogenous or exogenous origin (1) enter the alveolar space, infect it, and grow inside the alveolar macrophage (2). After necrotizing it, the bacilli infect neighboring alveolar macrophages and grow again, unleashing an inflammatory response that allows for the entry of monocytes and neutrophils in the lesions, and the drainage of bacilli *via* the lymphatic vessels (3). Once in the lymph node, the bacilli antigens are presented and a Th1 response is generated, resulting in the proliferation of specific effector lymphocytes that enter the systemic circulation toward the right heart. They reach the lung and are attracted to the infected lesions thanks to local chemokine production (4). The lymphocytes activate the infected macrophages, which kill most intracellular bacilli. A percentage of the bacilli become dormant and survive inside the necrotic tissue or the phagosomes of the macrophages (4a). Macrophages age and fill with lipid inclusions, turning into foamy macrophages, and are drained with the alveolar fluid toward the bronchial tree (6) entering the esophagus and reaching the gastrointestinal system where they are finally expelled from the body (7). Some of these foamy macrophages are destroyed on the way toward the pharynx, liberating the bacilli that become part of the aerosols and can reenter the lung again (1). This cycle is interrupted with the encapsulation and calcification of the granuloma, due to the action of the interlobular septa (5), or when neutrophils are attracted to the lesion, initially around the infected foamy macrophages, progressing toward a large degree of infiltration and cavitation (4b).

Twenty years ago, there was the tendency to consider granulomas as a sort of “bunker” where bacilli were kept enclosed. Currently, intravital imaging offers the possibility of obtaining real-time videos of the organization of these structures, showing their dynamics, with a constant movement of cells entering and leaving BCG-induced granulomas (23). In addition, the discovery of the formation of foamy macrophages in the granuloma and their drainage, infected with dormant bacilli, toward the bronchioles, explained the continuous reinfection and the constant growth of pulmonary infiltration in the murine model, while the bacillary concentration remained stable (24–26). This observation led to the “dynamic hypothesis” concept, based on the constant drainage from the granulomas (27). This process is driven by old macrophages that, after phagocyting the cellular debris of the intragranulomatous necrosis together with the mixed dormant bacilli, become foamy macrophages and are drained with the alveolar fluid *via* the mucociliary escalator. Thus, the majority of dormant bacilli are drained toward the gastrointestinal tract, but there is a small proportion that, after the destruction of foamy macrophages in the bronchi, infect the internal aerosols, enabling pulmonary reinfection (Figure 1). Recent data using positron emission tomography in the experimental non-human primate model (28) and in the evaluation of TB treatment in humans (29) have demonstrated this endogenous reinfection process.

This phenomenon is the basis for at least two well-known practices: TB diagnosis in children, by looking for bacilli in stomach aspirates, even when open lesions are lacking (30), and the current latent tuberculosis infection (LTBI) treatment. The latter is based on the administration of isoniazid for 9 months (31). This drug is only active against growing bacilli. So how can it display efficacy killing dormant bacilli, which are responsible for LTBI? The answer is that dormant bacilli are eliminated *via* the physiological drain, and what isoniazid does is avoid reinfection caused by infected internal aerosols. Overall, the complete drainage of LTBI lesions takes around 9 months, demonstrating the dynamic nature of granulomas (27).

#### The Relevance of Primary and Postprimary TB

Along with the massive chest X-ray surveillance programmes after the Second World War (32) came the concept of primary TB: the disease developed in childhood, spontaneously healed, and calcified and thus easily seen on an X-ray. Generally, primary TB showed a pattern with a nodule in the lower lobes and lymph node calcification. Postprimary (adult) disease was characterized by the presence of infiltration with or without cavitation in the upper lobe, without affecting the lymph nodes. It was then speculated that postprimary TB was the consequence of the reactivation of a secondary seeding of *Mycobacteria* that escaped *via* the blood circulation from the primary lesions in the lower lobe during childhood. This theory (the “Unitary Concept”) also considered that, once infected, subjects were always infected and protected against external reinfection (33).

The analysis of the relationship between recently acquired and remotely acquired pulmonary TB, clinical and demographic variables, and radiographic features by using molecular fingerprinting and conventional epidemiology has proven that adults can develop upper lobe infiltration—cavitation as a consequence of a recent infection and that the radiological pattern is related to the immune status of the patient and not to the time lapse after infection (34). Radiologically speaking, today, we define two patterns: the atypical one, when the lower lobes plus the lymph nodes are affected, which is seen in immunosuppressed persons, and the typical pattern, observed in immunocompetent persons who display upper lobe infiltration without the lymph nodes being affected.

### What We Have Missed

#### *M. tuberculosis* Can Always Infect and Reinfect Us, Regardless of Our Immune Status

So far, up to 13 vaccine candidates are already in the clinical pipeline (35). None of them have ever proven the capacity to avoid *M. tuberculosis* infection (36). Such a demonstration would represent the greatest scientific achievement in the twenty-first century. But it will not happen.

*Mycobacterium tuberculosis* infection takes place in the alveolar macrophage. This cell specializes in keeping the alveoli clean and is constantly infected. Fortunately, this cell does not recruit help from the immune system every time it is infected, otherwise our lungs would be constantly inflamed and the gas exchange function seriously hampered. The special anatomy of the alveolar space also has to be considered. Alveoli are sealed from the bloodstream to preserve the correct surface tension and avoid their collapse. Therefore, the presence of antibodies in this space is impossible, and an antibody-based immune response cannot avoid infection (37).

Moreover, the growth of *M. tuberculosis* is quite peculiar. It is slow, with a doubling time of around 24 h, and it blocks the macrophage apoptosis mechanism (38). This means that the bacilli can benefit from the internal milieu of the macrophage for growing until they cause its necrosis. As the maximum capacity of a human alveolar macrophage is around 60 bacilli, necrosis after infection by only 1 bacillus takes place around 6 days after its phagocytosis (39). Once the first macrophage has been destroyed, the bacilli infect the neighbor alveoli, aided by the constant movement of the lung, and the process starts all over again. However, after the destruction of several macrophages, there is enough chemokine production to induce an inflammatory response, drain the bacilli, and trigger the immune response in the lymph node.

This immune response is based on the proliferation of specific Th1 lymphocytes in the lymph node. It can happen quicker if there are already specific memory lymphocytes but, in any case, those cells have to locate the lesion, and they will be attracted to it only if a big enough inflammatory response is generated (40, 41).

All in all, this means that alveolar macrophages can be continually infected, even when specific effector lymphocytes are available, because they require a local inflammatory response strong enough to attract them (42).

In this regard, it is well known that the higher the TB incidence in a region, the higher the reinfection rate its inhabitants suffer and the greater the chance of TB induction (43). This is a very important point. Traditionally, disease induction caused by reactivation has been overemphasized to the point that vaccine efficacy is only tested against one challenge. High-incidence areas need a vaccine able to offer protection against multiple reinfections. This is obvious. In fact, it is probably the most important reason why BCG does not work in several geographic areas (42), and there is a simple explanation. As mentioned earlier, *M. tuberculosis* infection itself induces a protective immune response equal to the one elicited by BCG (13). Therefore, BCG vaccination only provides an advantage against the initial *M. tuberculosis* infection, by eliciting a faster immune response. However, in the context of several reinfections, there is no such advantage, as reinfection itself induces the immune boost, so there is no advantage of being BCG vaccinated. Of course other factors might also play a role, like coinfection with parasites (44) or environmental mycobacteria (45), together with individual genetic susceptibility (46). On the contrary, what seems to be unrelated is the varying virulence exhibited by specific *M. tuberculosis* strains (47).

#### Size Matters: The Role of Interlobular Septa

Standard medical practice for TB diagnosis entails the tuberculin skin test or the interferon gamma release assay to determine if a person is infected with *M. tuberculosis*. In case of positivity, the next step is a chest X-ray to look for lesions in the lungs. TB lesions can be distinguished in the lung with this technique once they reach a diameter of 10 mm (48).

Traditionally, when attempting to illustrate the progression from infection to disease, the size of TB lesions in both cases was shown to be the same (49); only the bacillary bulk harbored in each of them varied. This could be anecdotal, but it has certainly influenced many researchers.

**Figure 2:**
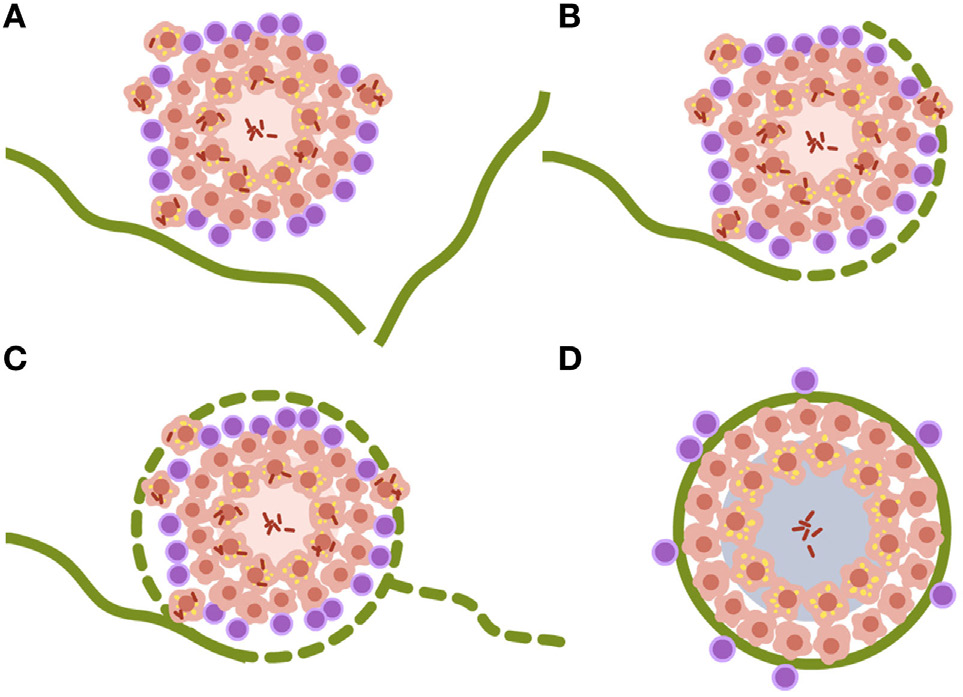
**Encapsulation process of *Mycobacterium tuberculosis* lesions**. (**A–D)** Interlobular septa are represented in green. Once the lesion is contacted, the encapsulation process starts surrounding it. The capsule completely envelops the lesion and aids the organization of miofibroblasts that stabilize the lesion. Non-drained necrotic tissue finally becomes calcified. Data are obtained from Gil et al. (51).

Looking at the progression of lesions in large mammals, infection lesions are not bigger than the ones we see in mice, around 0.5–1 mm at the time the immune response appears, 3 weeks after challenge (25). The major difference is that large mammal’s lungs are structured in cubes of around 1 cm^3^ surrounded by a collagen bag, the interlobular septum. These septa make up a net that conveys the forces induced by the movement of the diaphragm to inflate the lung, without tearing the delicate structure of the parenchyma designed for gas exchange (50). Fibroblasts in the septa can detect tiny lesions in the parenchyma, even those with a diameter of less than 1 mm. Once detected, they encapsulate them within 1–2 weeks, as has been demonstrated in the TB experimental model in minipigs (51) (Figure 2).

**Figure 3:**
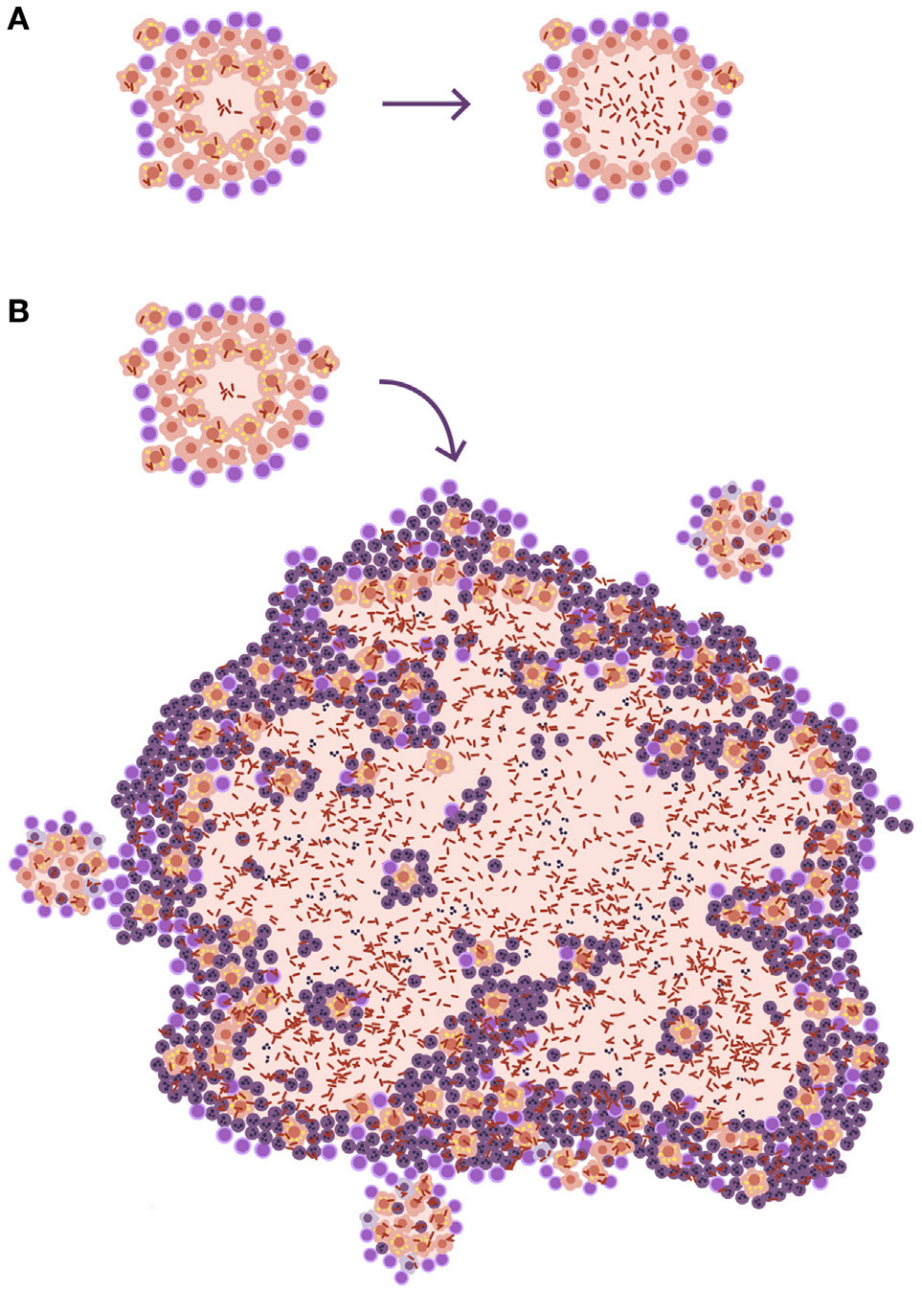
**Size matters in progression toward tuberculosis (TB)**. **(A)** Disease development as it is usually represented in publications. The only difference shown is that, in the lesion corresponding to disease, there is a higher bacillary concentration. **(B)** A more realistic approach. The TB disease lesion is exudative, with a predominance of neutrophilic infiltration together with massive necrosis, and it is much larger than the infected lesion. It must be taken into account that lesions need to be at least 10 mm in diameter to be visible on a chest X-ray and that infection lesions measure between 0.5 and 2 mm.

The size of lesions in disease should be depicted at least 10 times larger than the infected ones, to aid understanding of the process (Figure 3). In fact, one of the most important questions when addressing the issue of the progression from infection to disease should be how can a lesion of less than 1 mm become a 10 mm one. Especially taking into account the special circumstances found in the human lung, where the encapsulation process is so efficient.

Precisely why the encapsulation process has merited so little interest in the TB field should be questioned, seeing how important it is for interrupting bacillary drainage, ensuring the infection remains latent, and avoiding progression from infection to disease.

#### Neutrophils Are Key to Disease Development

Neutrophils are cells associated with acute infectious disease, mainly by extracellular pathogens (52), and linked to explosive inflammatory reactions that develop over the course of hours and usually end with the destruction of the infiltrated tissue, which fills with apoptotic or necrotic neutrophils, producing pus.

*Mycobacterium tuberculosis* is a slowly growing intracellular pathogen. This is the reason why, for a long time, and especially over the last 20 years, the role of neutrophils in TB has not been considered. This is clearly illustrated by the virtual models that have been built: neutrophils are not even mentioned (53).

**Figure 4:**
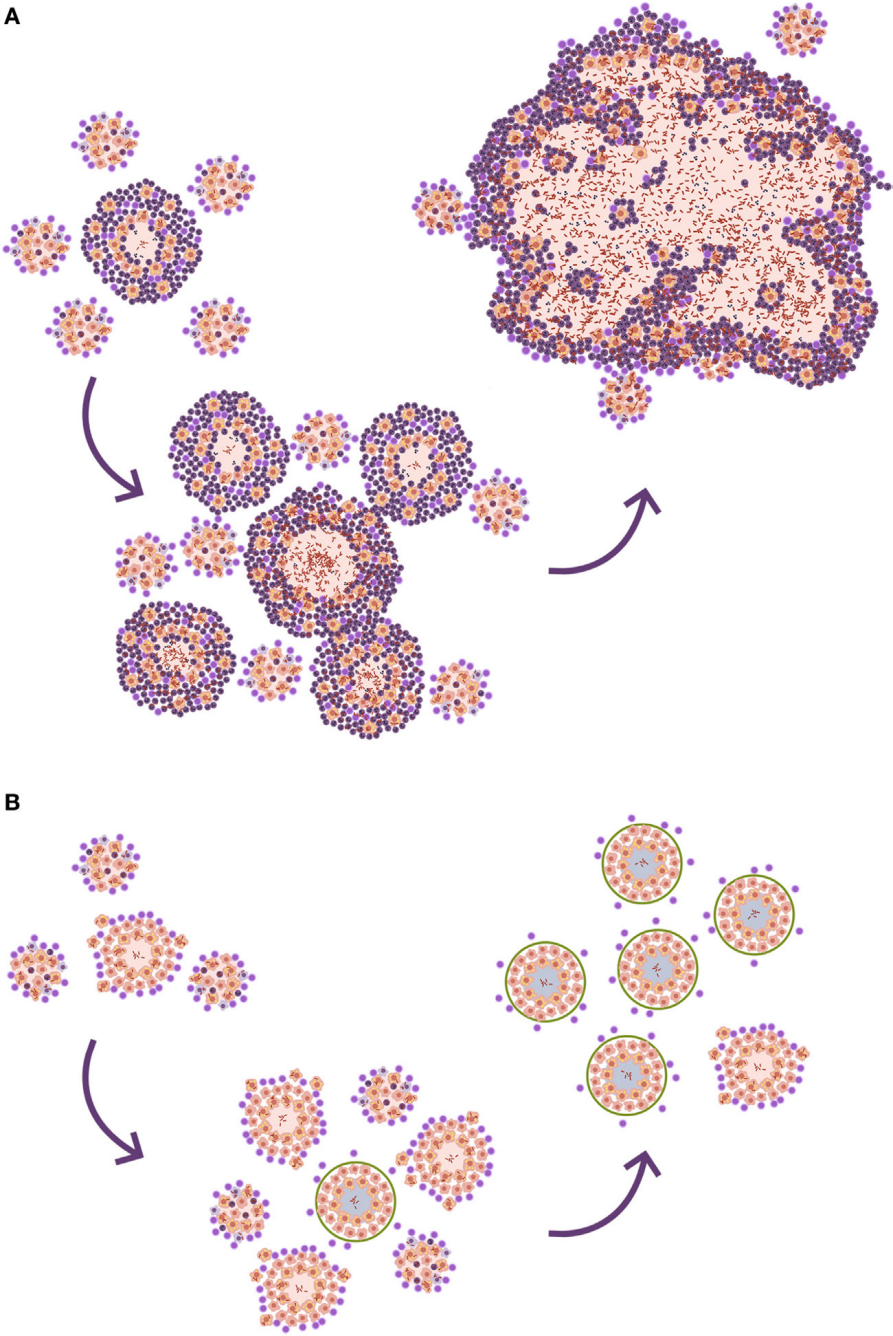
**Evolution toward active tuberculosis (TB)**. **(A)** The process toward a neutrophilic infiltrated lesion favors the induction of new lesions and their rapid growth; this leads to lesion coalescence and the induction of an active TB lesion. On the contrary, **(B)** shows a well-controlled lesion, without neutrophilic infiltration, which hardly induces new lesions and soon becomes encapsulated, blocking the induction of active TB.

Looking at the TB murine model, the authors have observed the presence of neutrophils during the acute phase, inducing tiny infiltrates, and have even related their presence to the presentation of antigens to the lymph nodes, but mainly as a limited phenomenon. This limited role is observed in all inbred mice but one: the C3HeB/FeJ. Ten years ago, it was observed that C3HeB/FeJ mice infected with *M. tuberculosis* surprisingly developed massive intragranulomatous necrosis (54). Further research was conducted to ascertain if this necrosis was linked to liquefaction, in which case, the model could be considered a “human-like” TB disease model. Consequently, the evolution of the lesions was carefully followed. The results showed how the size of the lesions increased rapidly, in a matter of days, thanks to peripheral growth linked to infected foamy macrophages heavily surrounded by neutrophils. From these original lesions, new ones appeared, and they all coalesced to induce massive lesions (55). This process illustrated how a lesion of less than 1 mm could become a 10 mm one, avoiding the encapsulation processes seen in large mammals. The process takes place in around 10 days (Figure 4). A parallelism can be drawn with the way soap bubbles are formed, and this has aided its modeling, becoming known as the “bubble model” (56).

At the beginning of the twentieth century, authors demonstrated that TB patients present two kinds of lesions: proliferative and exudative. The former are well structured and controlled, based on the presence of epithelioid cells and lymphocytes, like the ones developed by infected mice. The latter are mainly infiltrated with neutrophils and related to the expansion of the lesions and the induction of cavitation and liquefaction (57). A recent review on human pathology run in parallel by Hunter, searching through a wider selection of authors, has led to the same conclusion (58). In addition, the role of neutrophils has recently been reinforced by the data obtained from a search for a biosignature for TB progression (59, 60).

**Figure 5:**
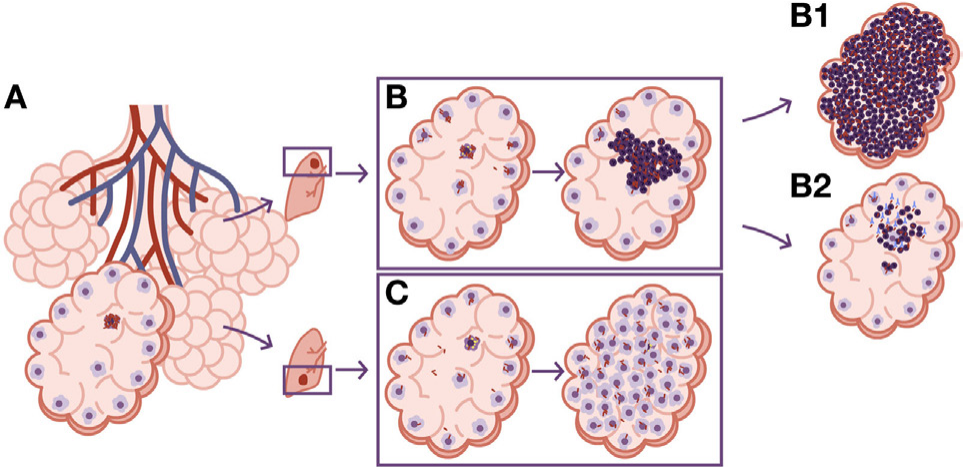
**Differences in local drainage determine the nature of the lesion**. **(A)** The necrosis of an alveolar macrophage releases intracellular bacilli. The rate of bacilli drainage follows the breathing amplitude of the lobe, which is lower in the upper lobes, resulting in lower bacillary drainage **(B)**. As a result, new incoming macrophages must face higher bacillary concentrations that, once the inflammatory response is triggered, cause a predominance of neutrophilic infiltration and the induction of neutrophilic extracellular traps that favor extracellular bacillary growth. This is the perfect scenario for the protective role of circulating antibodies, which can neutralize the bacilli and reduce the inflammatory response, controlling progression toward TB disease. **(B1)** The progression without antibodies. **(B2)** The control achieved by the presence of neutralizing antibodies. On the contrary, the larger breathing amplitude in the lower lobes causes significant bacillary dissemination, so macrophages face lower bacillary loads, favoring a less inflammatory lesion dominated by macrophages (**C**).

Tuberculosis tropism in the upper lobes can be also explained by neutrophilic infiltration. Upper lobes tend to mobilize less than the lower ones, simply because that is the region where the lungs “hang,” while the lower region is more directly influenced by the diaphragmatic contraction. The lack of mobilization in the upper lobes causes lower blood circulation and lower gas exchange, leading to an increase in oxygen pressure that had traditionally and erroneously been linked to higher bacillary growth (61). What is relevant about the lack of mobilization in the upper lobes is that bacilli tend to accumulate locally after the destruction of alveolar macrophages. This accumulation means that new, incoming macrophages have to phagocyte higher bacillary concentrations than in the lower lobes. This in turn causes more necrosis and the attraction of neutrophils that lead to the rapid increase in the size of the lesion (Figure 5). This fact also induces a Th17 immune response that stabilizes the neutrophilic infiltration at that site (62).

In this regard, it must be assumed that some genetic polymorphism must determine a more intensive reaction to this challenge, thus explaining why certain families were more prone to suffer TB than others. Unfortunately, precisely which polymorphism promotes this exaggerated inflammatory response has not been yet clarified, but it would certainly signify a valuable improvement in the field.

#### The Role of Antibodies in Progression toward Disease

The presence of antibodies does not offer protection against *M. tuberculosis* infection, because they cannot enter the alveolar space. However, the study of progression of infection in C3HeB/FeJ mice has demonstrated that neutrophilic infiltration builds neutrophilic extracellular traps that favor the extracellular growth of bacilli (63). In this phase, the presence of antibodies might certainly help to stop the progression of the lesion (Figure 5), enabling the encapsulation process and avoiding progression toward disease.

Several authors have proven the protective effect of antibodies. The article that reflects this process best investigated the protective effect of passive transference of *M. tuberculosis*-specific antibodies in a model of TB reactivation in SCID mice. The results clearly showed a protective effect, with a threefold reduction in lung infiltration and a 2-log reduction in pulmonary bacillary load (64).

Unfortunately, there is little information on the protective role of antibodies in humans. Only two projects have linked their level to disease prognosis: the case of antibodies against lipoarabinomannan and against the 38-kDa antigen, while the presence of IgG against Ag85A has been related to a reduced risk of developing TB disease in a case–control study in infants (65). Recently, an antibody-related signature has been identified for latent-infected or active TB patients, where glycosylation plays a key role (66).

#### Should We Tolerate *M. tuberculosis*?

**Figure 6:**
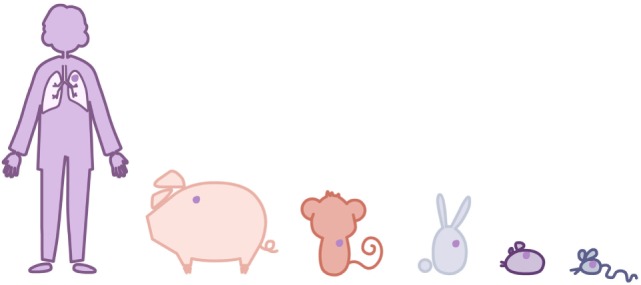
**Relationship between the volume of a cavity and the whole-body mass of a host**. Several laboratory animals plus humans are represented, comparing their mass with that of a cavitation. This is to illustrate the fact that in mice they would hardly develop, because they would disappear. Unable to eliminate the bacillary load, the majority of mouse strains (except C3Heb/FeJ) tolerate the presence of a constant, relatively high bacillary concentration in their lobes, controlling the induction of an excessive inflammatory response.

Why do a minority of patients infected by *M. tuberculosis* develop TB disease? As Figure 6 shows, mice will never develop lesions of the same magnitude as humans. The difference resides in the fact that although both bacilli and host cells have the same size; humans have a higher number of cells so, potentially, a certain amount of tissue can be destroyed without hampering our health status, at least more than in the case of mice.

Traditionally, mice have been considered “resistant” to *M. tuberculosis* infection because they show prolonged survival, usually quite similar to that of non-infected mice. In guinea pigs, considered susceptible, the contrary happens, because they generally die within weeks of infection. However, if the bacillary load is examined, it is found to be very high in mice compared to guinea pigs. This means that mice are not resistant, but tolerant to the infection, because they do not control the bacillary load, but it does not hamper their health status (67).

**Figure 7:**
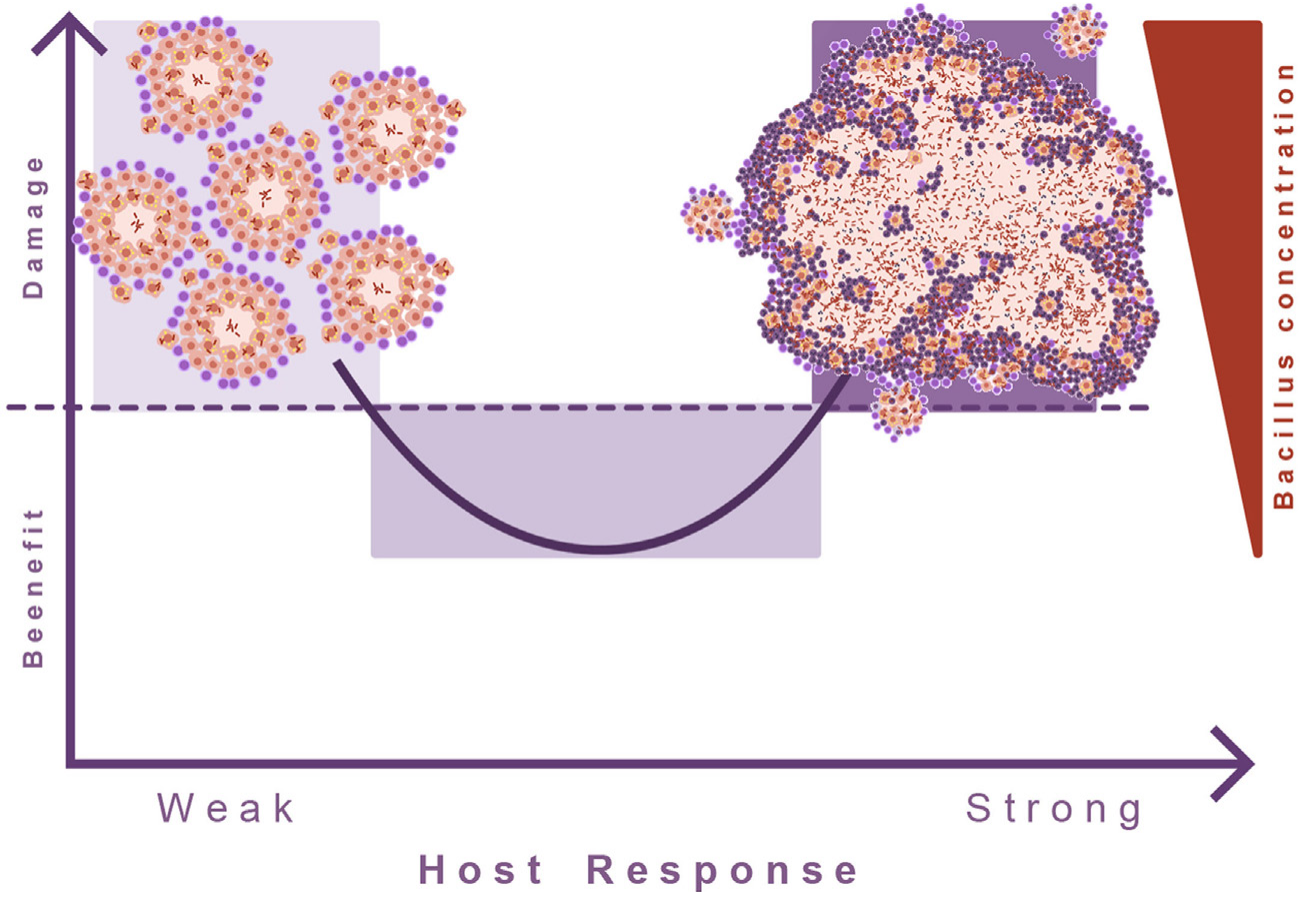
**Damage theory of infectious diseases**. This picture illustrates Arturo Casadevall’s “damage theory” (68). In the case of TB, a weak immune response leads to the proliferation of lesions and dissemination of the bacilli. However, the induction of an excessive inflammatory response also leads to exudative lesions characterized by massive tissue destruction. In this regard, we can consider that TB develops two kinds of diseases, and in between there is a wide range of situations where the immune response is balanced and allows the host to live with the bacilli without hampering its health status.

This led to the theory of the “damage-response” framework of microbial pathogenesis, which classifies all infectious diseases according the virulence potential of the pathogens and the response of the host to the pathogen. In the case of TB, the response against *M. tuberculosis* is a double-edged sword, advocating the need for a balanced immune response. Both a too weak or too strong immune response can cause active TB (68). It appears that in TB this concept has been poorly addressed. Invariably, induction of TB disease is linked to some sort of immunosuppression, but the consequences of an exaggerated immune response are poorly understood (Figure 7).

The relationship between a greater immune response and progression toward TB disease has recently been determined by an increased frequency of activated HLA-DR+ CD4+ T cells being linked to a heightened risk of TB disease (69). This higher immunological activation is probably originated by intercurrent infections like those caused by cytomegalovirus (CMV). Similarly, one of the major risks fueling TB is being overweight, which is related to type II diabetes (70), and in turn to a systemic pro-inflammatory response (71).

The work by Green et al. in the cynomolgus macaque model demonstrated that the animals that did not progress toward TB disease had significantly higher frequencies of Tregs in peripheral blood prior to infection, compared to macaques that developed active disease, supporting the idea that more Tregs prior to infection correlates with a better infection outcome (72). This made others consider protection against TB the consequence of a balanced immune response, able to stimulate a Th1 immune response but without causing too much damage, especially without causing excessive neutrophil infiltration (67, 73). In this regard, the proof of concept that a decreased inflammatory response causes a reduction of the bacillary load was experimentally demonstrated in a TB murine model after treatment with ibuprofen (74), a clear indication of the new approach towards the use of host-directed therapies in TB (75). Following this logic, a promising strategy to stop progression toward TB disease appears to be the induction of *M. tuberculosis* protein purified derivative-specific memory Tregs to counterbalance the Th17 (76–78). The role of Tregs has been controversial for a long time, as it was related to counterbalance Th1 response (79). This concept was progressively refined, placing Tregs in a more neutral role, without increasing the bacillary load in lesions at all (80), or even a protective one (81, 82). The fact is that the models that were used did not develop human-like lesions, and therefore, the role of pathology and exudative progression was not explored. When this parameter is included, as in C3HeB/FeJ mice (55) or NHP (72), Tregs response is clearly beneficial and is related to the interruption of progression toward active TB.

In conclusion, after 30 years of investing heavily in “bacilli-oriented therapies” to destroy *M. tuberculosis*, there now seems to be a new opportunity to address the control of TB epidemics using “host-oriented therapies,” which would enable a physiological approach to living with the presence of this bacillus: simply tolerating it!

#### One World, Ecology, Coinfection

Studies in wild-type animals have highlighted the role of coinfection in TB. In this case, it is important to note the impact of parasitosis as a source of Tregs response, balancing the immune response and thus protecting against progression from infection to disease. This concept must be approached cautiously as, so far, parasitosis has been related to a neutral (83, 84) effect in humans with regard to progression toward TB disease. On the contrary, concurrent infections such as CMV are potent immunity activators favoring progression toward TB (85, 86).

## TB is a Complex Infection: The Quest for the “Pink Swan”

Tuberculosis is the consequence of a major, complex process and, even though we have accumulated a vast amount of information and knowledge about it, we desperately need new tools and novel, groundbreaking concepts to aid us in the search for more efficacious strategies to curtail TB progression.

As a consequence of a lack of resources, TB researchers tend to organize in synergistic, monolithic consortiums to act coordinately and obtain funding. This is a positive and intelligent organizational set up, but it can be potentially deleterious from the intellectual point of view, as it can lead to homogeneous thinking.

It has been claimed that “out of the box thinking” is needed and that we are seeking for a “pink swan” (9): a unique and revolutionary concept that will have a massive impact in the field. The recruitment of all research groups under one single strategy may be incompatible with reaching this target. Large organizations need to harmonize to seek consensus. This is very important to complete file-intensive projects, requiring many samples, many subjects, etc. such as clinical trials. However, it probably is not that convenient when searching for new concepts, blue-sky thinking, because unifying, harmonizing exercises tend to have too great influence on the group, and marginalize divergent approaches.

In this sense, the fresh approaches to old methodological systems for testing new vaccines are very interesting. Mycobacterial Growth Inhibition Assay (MGIA), to ascertain mycobactericidal capacity, in spite of not yet understanding the mechanism of action (87) and the use of the monocyte:lymphocyte ratio in peripheral blood to predict the induction of TB disease are two clear examples of novel, imaginative ways of looking for a protective surrogate marker (88).

In a nutshell, it is necessary to encourage the creation of instruments to fund “free-thinking” concepts to advance TB research. In particular, it would be interesting to recruit knowledge from other disciplines, with fresh concepts and new ideas, to drive innovative projects capable of leading us to our “pink swan.”

## Author Contributions

P-JC is responsible for the whole review. This is an inaugural article.

## Conflict of Interest Statement

P-JC is one of the funders and the CSO of Manremyc, a company devoted to the oral development of the administration of heat-killed *Mycobacterium manresensis* as a way to induce low-dose tolerance and reduce the risk of the progression toward TB disease.
